# Case of Supposed Fungus Hæmatodes, Combined with Melanosis

**Published:** 1844-02-10

**Authors:** John J. Reese

**Affiliations:** Physician to the Philadelphia Dispensary


―――
### THE MEDICAL EXAMINER,

### And Record of Medical Science.

Vol. VII.] PHILADELPHIA, SATURDAY, FEBRUARY 10, 1844. [No. 3.

To the Editor of the Medical Examiner.

*My dear Sir*,—Enclosed I send you the notes of a case of some interest, which occurred a short time since in my practice. If you deem it of sufficient importance to present before your readers, it is entirely at your service.

Very respectfully and truly yours,

Jno. J. Reese.

301 *Walnut st.*, *Feb.* 3, 1844.

### CASE OF SUPPOSED FUNGUS HÆMATODES, COMBINED WITH MELANOSIS.

By John J. Reese, M. D.

Physician to the Philadelphia Dispensary.

F. B. aged 83 years, an American by birth, of an excellent constitution, had enjoyed uninterrupted health until about fifteen months since. In the early period of his life he had served in the army, and until very lately he followed the occupation of a shoemaker.

The first appearance of disease manifested itself a little more than a year ago, in the shape of a small tumour upon the upper posterior portion of the right shoulder, which from his own account, I suppose to have been an enlarged indurated lymphatic gland. A year previous, the patient had observed a tumour similar in appearance to this, (according to his own description) at the inner canthus of the right eye, which he called a *wart*, and which disappeared spontaneously about the time that the tumour on the shoulder showed itself. The latter tumour continued gradually to increase in size; and the lymphatic glands in the immediate vicinity also enlarged, several of them coalescing with the original one, and forming a knotted, tuberculated tumour. About four months ago, this took on ulceration,—probably in several places, as I judged from its appearance—discharging a large quantity of thin serum, and at times sero-purulent matter. Up to this period, the patient's general health remained perfectly good, his strength being yet sufficient to enable him to walk several miles a day. Now, however, his health commenced declining—and he began to be enfeebled by the amount of matter discharged. He applied at the Philadelphia Dispensary for relief, in November last, not having previously been under any medical care. Dr. Page, one of my colleagues, who first took charge of the case, applied mild escharotics to the ulcerated tumour, under the use of which, several small pieces of the fungous mass sloughed off. The patient also took the Iodide of Potassium, which however was discontinued, as it did not agree with his stomach. He came under my care in the early part of December, and presented the following appearance:—Emaciation not greater than usual for a person of his advanced age; strength much enfeebled; appetite impaired; bowels habitually costive; tongue nearly natural; pulse rather weak. The tumour on the shoulder consisted of a flattish fungous mass, about four and a half inches long, by three broad, and elevated about an inch above the level of the skin. It was of a dark liver-color, mottled with greyish and livid spots—full of foramina, and resembling very much a honey-comb. Its texture appeared to be tough and fibrous. It was not at all painful on pressure, nor did the patient complain of pain in it at any other time. The discharge from it was thin, yellowish, and sometimes mixed with blood, and so profuse, as completely to saturate his linen and even to pass through the bed clothes. In fact he complained *only* of the discharge, which he said was weakening him very rapidly. The lymphatic glands in the neighborhood were much enlarged—particularly one, of a dark color, near the edge of the tumour, and also the glands of the axilla of the affected side.

The patient had been using a generous diet, which was continued, together with quinine and the tonic tinctures. A powder, composed of burnt alum and chloride of lime, was sprinkled upon the tumour once a day, as much for the purpose of correcting the fetor, as to destroy, if possible, the fungous growth. An operation at his period of life, and in his enfeebled condition, was, of course, inadmissible. He continued, however, rapidly to decline, and towards the end of the month he was seized with a severe bronchitis, attended with a hard cough, sore throat, and pain of the right side, with great increase of feebleness. For this he was blistered, and took stimulant expectorants with carbonate of ammonia and morphia, and also wine-whey. This treatment appeared slightly to relieve him; but in the course of a few days he expectorated gangrenous matter and became exceedingly weak, being scarcely able to expectorate. He died on the 2d of January. For the two weeks previous to his death, his mind appeared to become somewhat imbecile; he would refuse to take his medicine, or even proper nourishment, unless compelled to do so.

The *Autopsy* was made twenty-four hours after death. I was kindly assisted in it by Drs. Parrish and Page, and Dr. Cunningham, of Alabama. The following appearances presented:

Body not much emaciated; surface studded with small hard tubercles in the course of the lymphatics, varying from the size of a pin's head, to that of a hazel nut; being larger near the tumour, and diminishing as the distance increased; disappearing altogether at the pelvic region. The glands of the axilla much enlarged, particularly the right. The tubercles following the course of the lymphatics of the upper extremities, very distinctly marked. On cutting into one of these superficial tubercles which seemed to be immediately beneath the skin, it was found to consist of a soft, dark chocolate coloured matter—apparently of a homogeneous character. The larger of the superficial tubercles exhibited this dark colour shining through the skin; the smaller ones did not, being scarcely raised above the surface.

The tumor was first removed. It was at one point firmly adherent to the subjacent muscle, being in fact identified with its structure; the rest of it was easily dissected off. It was of a darker color and more shrunken appearance than before death. There was also considerable ecchymosis for some distance around it, owing no doubt to the gravitation of the blood. The fungous mass, on being cut into, exhibited a uniform homogeneous appearance. There was no evidence of striæ or bands dividing it off into segments, as in the true cancerous tumours. The glands of the right axilla were very much enlarged, presenting a tumour of the size of a walnut. On removal, this was found attached to several others of nearly equal size, and extending deep in towards the thorax. These glands appeared to be entirely converted into melanosed matter, presenting the same dark appearance and consistence as the superficial gland. One of these contained a cyst lined by a membrane, and containing a semi-fluid matter.

The *cavity of the thorax* contained about two pints of bloody serum. There were pretty strong adhesions between the upper part of the *left* lung and pleura—none elsewhere. The lungs themselves were generally of a blacker colour than natural, and exhibited small masses of melanotic matter scattered throughout, about the size of a large pea; rather more numerous in the right lung than in the left, and in the upper than lower lobes. They were for the most part quite superficial, being directly under the pleura. The lower lobes exhibited marks of congestion, but no hepatization. There were no tubercles in either lung.

The *heart* appeared perfectly healthy, with the exception of some slight thickening of the walls of the left ventricle, and ossific deposits about the aortic valves; not however beyond what might be expected in so aged a subject. The *aorta* for some inches down, was converted into a hard cartilaginous tube; its inner surface exhibiting numerous deposites of bony matter.

The *liver* was the organ most involved in the melanotic disease. It was much enlarged, particularly in its perpendicular diameter, and would probably have weighed from eight to ten pounds. The whole organ was one complete mass of disease; nodules, about the size of a filbert, projecting out on every side, giving to the gland the feel of a large mass of knots. On making incisions, the entire organ was found studded throughout with these almost black masses, many of them containing little cysts precisely like those in the glands of the axilla, presenting a perfectly marbled appearance.

The *gall-bladder* contained a small quantity of dark viscid bile.

The *spleen* was about the usual size—if any thing, smaller than common. It consisted of a thick fibrous sac, enclosing a soft pultaceous mass, and several small cysts contained within it, each of which seemed to be lined by a membrane.

The *kidneys* presented nothing abnormal.

The *stomach* was healthy. The *intestines* were not examined, but, on their peritonæal coat, they afforded no appearances of disease.

The *mesenteric* glands were enlarged, but not so much involved as might have been expected.

The *pancreas* was not affected; but its *duct* was enlarged and completely converted into a bony tube. The rest of the body not examined.

*Remarks.*—I have detailed the foregoing case as affording an interesting example of what was supposed to be an external fungus hæmatodes, and general melanosis. As regards the *latter* disease, it was sufficiently evident on inspecting the body after death. As is generally the case, the melanotic deposite was most abundant in the liver; nearly the whole of that organ, as we have seen, being converted into it. Yet with all this mass of disease, the *functions* of the gland appeared to be but little affected. There was some constipation—but not more than is usual in aged persons; there was also a very slight icterode appearance of the conjunctivæ. This is an interesting fact, as it proves, among many others, the perfect independence of the different organic cells which enter into the composition of an organ—each cell being, in truth, an epitome of the whole gland, and, as such, performing all its functions.

The severe pain, which the patient experienced on the right side a few days before his death, might have been caused by the diseased liver. From the cough and difficulty of breathing attending it, I had supposed it an acute attack of pleurisy combined with bronchitis, and treated it as such. It was difficult to percuss or auscult the patient, from his excessive weakness.

As regards the tumour on the shoulder, it was the impression of most of those who saw it before death, that it was either fungus hæmatodes, or something between this and true cancer in a state of ulceration. On examining it, however, after death, I had some doubts as to the true nature of its composition. In many respects, it is true, it resembled a malignant fungus—in others, again it looked more like a melanotic tumour exterior to the skin. Its internal structure coincided very much in appearance with the masses of that substance seen in other parts of the subject, only that it was much more condensed. Certainly melanosis may attack the skin as well as any other tissue of the body; and it is known that a fungus does sometimes sprout from the part where this dark deposite has taken place. From what I can learn, however, such a form of it is very rare. It may also be remarked that both melanosis and fungus hæmatodes may co-exist in the same individual.

I shall be much gratified if the foregoing notes should elicit from some of your correspondents additional information, and cases of *external* melanosis assuming the fungous or ulcerated form.

It is generally supposed, that this deposite, unlike either cancer or tubercle, is very little, if at all, disposed to undergo softening.

